# Streptococcus pyogenes Is Associated with Idiopathic Cutaneous Ulcers in Children on a Yaws-Endemic Island

**DOI:** 10.1128/mBio.03162-20

**Published:** 2021-01-12

**Authors:** Brad Griesenauer, Camila González-Beiras, Katherine R. Fortney, Huaiying Lin, Xiang Gao, Charmie Godornes, David E. Nelson, Barry P. Katz, Sheila A. Lukehart, Oriol Mitjà, Qunfeng Dong, Stanley M. Spinola

**Affiliations:** a Department of Microbiology and Immunology, Indiana University School of Medicine, Indianapolis, Indiana, USA; b Carretera de Canyet, Hospital Universitari Germans Trias i Pujol, Badalona, Barcelona, Spain; c Department of Medicine, Stritch School of Medicine, Loyola University Chicago, Maywood, Illinois, USA; d Department of Medicine, University of Washington, Seattle, Washington, USA; e Department of Global Health, University of Washington, Seattle, Washington, USA; f Department of Biostatistics, Indiana University School of Medicine, Indianapolis, Indiana, USA; g Richard M. Fairbanks School of Public Health, Indianapolis, Indiana, USA; h Department of Medicine, Indiana University School of Medicine, Indianapolis, Indiana, USA; i Department of Pathology and Laboratory Medicine, Indiana University School of Medicine, Indianapolis, Indiana, USA; Rutgers University

**Keywords:** *Haemophilus ducreyi*, *Streptococcus pyogenes*, *Treponema pallidum* subsp. *pertenue*, cutaneous ulcers, microbiome

## Abstract

Exudative cutaneous ulcers (CU) in yaws-endemic areas are associated with Treponema pallidum subsp. *pertenue* (TP) and Haemophilus ducreyi (HD), but one-third of CU cases are idiopathic (IU). Using mass drug administration (MDA) of azithromycin, a yaws eradication campaign on Lihir Island in Papua New Guinea reduced but failed to eradicate yaws; IU rates remained constant throughout the campaign. To identify potential etiologies of IU, we obtained swabs of CU lesions (*n* = 279) and of the skin of asymptomatic controls (AC; *n* = 233) from the Lihir Island cohort and characterized their microbiomes using a metagenomics approach. CU bacterial communities were less diverse than those of the AC. Using real-time multiplex PCR with pathogen-specific primers, we separated CU specimens into HD-positive (HD+), TP+, HD+TP+, and IU groups. Each CU subgroup formed a distinct bacterial community, defined by the species detected and/or the relative abundances of species within each group. Streptococcus pyogenes was the most abundant organism in IU (22.65%) and was enriched in IU compared to other ulcer groups. Follow-up samples (*n* = 31) were obtained from nonhealed ulcers; the average relative abundance of S. pyogenes was 30.11% in not improved ulcers and 0.88% in improved ulcers, suggesting that S. pyogenes in the not improved ulcers may be azithromycin resistant. Catonella morbi was enriched in IU that lacked S. pyogenes. As some S. pyogenes and TP strains are macrolide resistant, penicillin may be the drug of choice for CU azithromycin treatment failures. Our study will aid in the design of diagnostic tests and selective therapies for CU.

## Abstract (precis)

Cutaneous ulcers (CU) affect approximately 100,000 children in the tropics each year. While two-thirds of CU are caused by Treponema pallidum subspecies *pertenue* and Haemophilus ducreyi, the cause(s) of the remaining one-third is unknown.

## INTRODUCTION

Exudative cutaneous ulcers (CU) afflict 5 to 15% of children in tropical countries in the South Pacific islands and parts of Africa. CU typically present on the lower legs at single or multiple sites and can lead to chronic disfigurement and disability (1). CU were originally attributed almost entirely to yaws, caused by Treponema pallidum subsp. *pertenue* (TP) (2). Recently, evidence has emerged that many presumptive cases of yaws were instead caused by Haemophilus ducreyi, the causative agent of chancroid (3). With ∼100,000 cases of yaws reported annually to the World Health Organization (WHO) and ∼100 million children at risk for infection (1, 4), the WHO has classified yaws as a neglected tropical disease (NTD) targeted for eradication.

Oral azithromycin was recently shown to be as effective as injectable benzathine penicillin to treat yaws (5). This finding prompted clinical trials of a WHO yaws eradication campaign using the Morges strategy, which includes diagnostic sampling of persons with CU in an endemic community, mass drug administration (MDA) of a single dose of oral azithromycin to the entire population of the community, and case finding and treatment of subsequent CU cases and their household contacts with azithromycin every 6 months (6). On Lihir Island in Papua New Guinea, prior to MDA, molecular diagnostic tests showed that ∼20% of CU cases contained TP DNA, ∼50% contained Haemophilus ducreyi (HD) DNA, ∼10% contained both TP and HD DNA, and ∼20% of CU contained neither TP nor HD DNA and are classified as idiopathic ulcers (IU) (3).

The initial results of the yaws eradication campaign 1 year after MDA were promising in that CU prevalence decreased from 10.2% to 1.7% and the TP DNA detection rates decreased from 21% to 11% (7). However, CU prevalence failed to decrease further, and the trial was halted 42 months after MDA due to failure to eradicate yaws. Several factors may have contributed to this outcome. First, TP DNA detection rates increased to 35% in part due to the emergence of azithromycin-resistant TP (8). Second, environmental reservoirs of HD were identified, including the skin of asymptomatic children, flies, and bed linens (9), which may explain the failure to decrease rates of HD in CU. Third, incomplete MDA coverage—only 83% of Lihir Island inhabitants received azithromycin—and importation from surrounding islands could have led to the persistence of HD and TP on the island (8). Finally, the overall rate of IU was 26% throughout the trial (8), suggesting that the potential pathogens that cause IU are resistant to azithromycin and/or there are environmental reservoir(s) for the agents that cause IU, similar to the environmental reservoirs for HD.

A better understanding of the microbiome of CU and the skin of asymptomatic children in endemic communities could identify new etiologies of CU and guide the development of diagnostic and environmental studies that will influence the clinical management of CU and future eradication efforts. Here, we used V1-V3 16S rRNA gene sequencing on CU and asymptomatic control (AC) swabs collected from the yaws eradication campaign on Lihir Island to compare the microbiomes of the CU and AC samples and to identify possible causative agents of IU.

## RESULTS

### Collection of specimens.

To initiate the yaws eradication campaign, a single oral dose of azithromycin was given to 83% of the entire population of Lihir Island, Papua New Guinea (7, 8). The islanders were then examined either in school or in their villages every 6 months for up to 42 months. Those who subsequently developed CU had their lesions swabbed, were treated with azithromycin, and examined 2 weeks later. As the trial was halted at 42 months, a limited specimen collection from those with CU was done at 48 months to monitor for azithromycin resistance in TP. We obtained swabs of CU from 106, 107, and 66 participants at 36 months, 42 months, and 48 months post-MDA of azithromycin, respectively, totaling 279 specimens. To collect these specimens, a sterile, dry, Dacron-tipped swab was vigorously rubbed at the base of the ulcer and inserted into an Eppendorf tube containing lysis buffer. Of the 279 participants, 31 had ulcers that persisted at the follow-up visit, and their ulcers were swabbed a second time. Persistent ulcers were categorized as “improved” or “not improved” based on ulcer bed size and presence of granulation tissue. Participants whose ulcers had not improved were retreated with azithromycin. We also collected swabs of intact skin on the lower legs from AC (*n* = 233) who came from either the same school or household as a CU case. We were unable to collect AC specimens for 46 CU participants. To account for possible environmental contamination, we collected environmental control swabs (EC) by placing a swab in a lysis buffer in the presence of each CU (*n* = 234) or AC (*n* = 233) participant without touching the participant. We were unable to collect EC specimens for 45 participants who had CU. A summary of the samples and the demographic data of the CU cases at each time point is in Table S1 in the supplemental material.

10.1128/mBio.03162-20.1 TABLE S1 Specimens and demographic information of children with ulcers. Download Table S1, DOCX file, 0.01 MB. Copyright © 2021 Griesenauer et al. 2021 Griesenauer et al. This content is distributed under the terms of the Creative Commons Attribution 4.0 International license.

### Identifying the etiology of ulcer specimens.

As reported previously, we used real-time multiplex PCR with pathogen-specific primers to identify CU specimens that contained TP, HD, both (TP/HD) DNAs, or neither DNA (IU) (8, 10). A positive test was defined as detection of TP, HD, or both TP/HD DNAs in at least one of four or five replicates. Specimens that did not test positive for these pathogens were concentrated, and PCR was again performed, with a positive test being similarly defined. Of the 279 CU specimens, 275 contained DNA for human β-globulin, which was used as a marker for DNA integrity as the specimens primarily contain host DNA. Of those 275 specimens, 88 (32%) were classified as TP, 87 (32%) as HD, 37 (13%) as TP/HD, and 63 (23%) as IU (Table 1).

**TABLE 1:** Number of ulcers by classification in the overall data set^*a*^

Etiology	No. of ulcers at the following time after MDA:			Total no. of ulcers^*b*^ (%)
	36 mo.	42 mo.	48 mo.	
TP	24	37	27	88 (32)
HD	48	30	9	87 (32)
TP/HD	12	14	11	37 (13)
IU	18	26	19	63 (23)

a Abbreviations: MDA, mass drug administration; TP, T. pallidum subsp. *pertenue*; HD, H. ducreyi; TP/HD, T. pallidum subsp. *pertenue* and H. ducreyi; IU, idiopathic ulcer.

b PCR results for four participants were excluded due to the absence of both pathogen and human β-globulin DNA in the ulcer swabs.

We performed 16S rRNA gene sequencing on a total of 1,037 specimens, consisting of 275 CU swabs, 233 AC swabs, 234 ulcer EC swabs, 233 asymptomatic control EC swabs, 31 follow-up swabs, and 31 follow-up EC swabs. After using the R package “phyloseq” to bioinformatically remove 16S rRNA gene sequences that corresponded to rare taxa (<200 reads/taxa present throughout specimens) and to remove specimens that yielded <500 reads/specimen, 897 specimens remained with a mean of ∼92,000 high-quality reads per specimen (Table S2). Two hundred sixty-five (96%) CU specimens remained after filtering. Of the 265 CU specimens, 83 (31%) were classified as TP, 85 (32%) as HD, 36 (14%) as TP/HD, and 61 (23%) as IU by PCR.

10.1128/mBio.03162-20.2 TABLE S2 Summary of sequencing results after pruning. Download Table S2, DOCX file, 0.01 MB. Copyright © 2021 Griesenauer et al. 2021 Griesenauer et al. This content is distributed under the terms of the Creative Commons Attribution 4.0 International license.

Although each of the pathogen-associated CU groups contained a high mean relative abundance (7.0% to 15.7%) of 16S rRNA gene reads of their respective pathogen(s), there were discrepancies in the results of the two assays. For example, of the 85 CU specimens classified as HD by PCR, only 63 (74.1%) contained HD 16S rRNA gene reads. Similar discrepancies were seen for the other three subgroups of CU (Table S3). Because the results of the two assays were discordant, we developed a stringent classification system. In this system, specimens that had >0.1% relative abundance and >100 reads of either HD, TP, or both by 16S rRNA gene sequencing and had ≥75% of wells show amplification by PCR for HD, TP, or both were classified as HD positive (HD+), TP+, or HD+TP+ specimens (Table 2). IU specimens were defined as those that had <0.1% relative abundance and <100 reads and were PCR negative for both pathogens. Specimens that did not meet all these criteria were excluded, leaving 157 (59%) stringently classified CU specimens. We examined both the stringent and overall (defined only by the pathogen-specific PCR results) data sets for possible confounding factors (age, gender, or sample acquisition time) affecting the classification of CU into TP, HD, TP/HD, or IU and found no confounding effects (data not shown). We performed all downstream analyses on both the stringent and the overall CU data sets. Except as is noted below, the results from the stringent data set and the overall data set were similar; data from the stringent data set are presented in the main text, and data from the overall data set are presented in the supplemental figures.

**TABLE 2:** Stringent classification criteria for CU^*a*^

Classification approach	TP	HD	HD/TP	IU
16S rRNA relative abundance	>0.1% TP	>0.1% HD	>0.1% HD and >0.1% TP	<0.1% HD and <0.1% TP
16S rRNA read count	>100 TP reads	>100 HD reads	>100 HD reads and >100 TP reads	<100 HD reads and <100 TP reads
% wells positive in PCR tests	≥75% for TP	≥75% for HD	≥75% for both HD and TP	0% for both HD and TP

No. of samples (*n* = 157)	51	41	17	48

a Abbreviations: qPCR, quantitative PCR; TP, T. pallidum subsp. *pertenue*; HD, H. ducreyi; HD/TP, H. ducreyi and T. pallidum subsp. *pertenue*; IU, idiopathic ulcer.

10.1128/mBio.03162-20.3 TABLE S3 16S rRNA sequencing results grouped by PCR classification. Download Table S3, DOCX file, 0.01 MB. Copyright © 2021 Griesenauer et al. 2021 Griesenauer et al. This content is distributed under the terms of the Creative Commons Attribution 4.0 International license.

### Removal of contaminants.

After processing our sequences through the DADA2 pipeline v1.8 (11), which infers exact amplicon sequence variants (ASVs) from high-throughput amplicon sequencing data and assigns taxonomic classification, 2,936 16S rRNA gene ASVs were identified in the CU stringent data set, 5,620 ASVs in the overall CU data set, and 14,644 ASVs in the AC group. We used ASVs rather than species or operational taxonomic units because this method can resolve single nucleotide polymorphisms, potentially permitting strain-level classifications. In addition, ASVs from this study can be directly compared to other studies using ASVs or shotgun sequencing.

The skin microbiome has a low bacterial abundance, and the results of 16S rRNA gene amplification and sequencing can be affected by environmental contamination. To account for this, contaminants were removed through a data enrichment step (12). We retained ASVs that had at least threefold-higher relative abundance in the CU or AC specimens compared to their respective EC specimens and assay controls and at least >0.01% relative abundance in the CU or AC specimens. The remaining ASVs were omitted from the data set. After the data enrichment step, 382 ASVs representing 70.0% of the total high-quality reads in the stringent CU data set (397 ASVs representing 60.1% of the total read count in the overall data set) and 900 ASVs representing 41.9% of the total high-quality read count in the AC group were retained.

### Ulcer versus asymptomatic skin microbiome.

**FIG 1:**
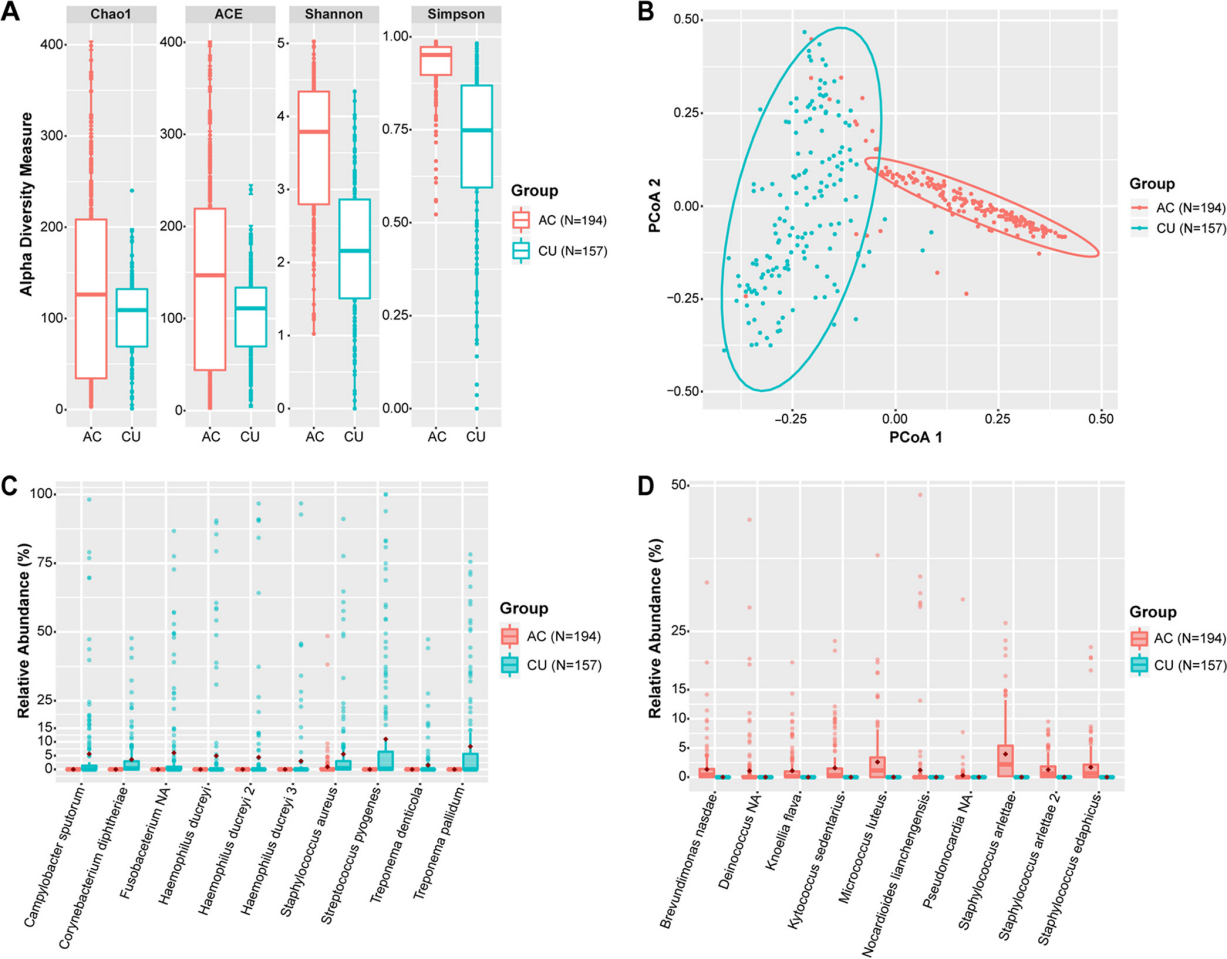
Ulcer vs skin microbiomes in the stringent data set. (A) Box plots showing Chao1 and ACE richness estimates and Shannon and Simpson diversity indices of ulcers (ulcers) and asymptomatic controls. Significant *P* values: Chao1, 0.043; ACE, 0.001; Shannon, <0.001; Simpson, <0.001. (B) PCoA of ulcer and asymptomatic control microbiomes. PERMANOVA *P* value = 0.001. (C) Box plots of top 10 ASVs found in ulcers and comparison of their relative abundances in ulcers and asymptomatic controls. All 10 comparisons have *P* values of <0.001. (D) Box plots of top 10 ASVs found in the asymptomatic controls and comparison of their relative abundances in ulcers and asymptomatic skin. All 10 comparisons have *P* values of <0.001. All box plots show medians with hinges corresponding to 25th and 75th percentiles and whiskers extending no further than 1.5× interquartile range from the hinges. Red diamonds signify means. Medians are shifted toward zero due to the absence of many of the ASVs in the majority of the samples. Abbreviations: AC, asymptomatic control specimens; CU, cutaneous ulcer specimens; NA, species not available.

To compare the microbiomes of CU to those of AC, we first examined differences in bacterial richness using the Chao1 and abundance coverage-based estimator (ACE) estimates and bacterial diversity using the Shannon and Simpson indices. Chao1 and ACE estimates attempt to quantify the number of unique microbial species, while Shannon and Simpson indices attempt to measure diversity of the microbiota by accounting for both number of species present and how abundant these species are in a sample. By each measure, we found that the CU microbiome was less rich and less diverse than the AC microbiome (Fig. 1A; see also Fig. S1A in the supplemental material). We assessed differences in community composition based on taxa present and their relative abundances with Bray-Curtis dissimilarities, which allow calculation of how alike or unalike specimens are, by using the abundance data from the 16S rRNA gene sequencing, and visualized these data using principal-coordinate analysis (PCoA). We found that that taxa in CU and AC were significantly different (Fig. 1B; Fig. S1B).

10.1128/mBio.03162-20.4 FIG S1 Ulcer vs skin microbiomes in the overall data set. (A) Box plots of Chao1 and ACE richness estimates and Shannon and Simpson diversity indices. Significant *P* values: Chao1, *P* < 0.001; ACE, *P* = 0.002; Shannon, *P* < 0.001; Simpson, *P* < 0.001. (B) PCoA of ulcer and asymptomatic skin microbiomes. PERMANOVA *P* value = 0.001. (C) Box plots of top 10 ASVs found in ulcers and comparison of their relative abundances between all ulcers and asymptomatic skin. All 10 comparisons have *P* values of <0.001. (D) Box plots of top 10 ASVs found in asymptomatic skin and comparison of their relative abundances between all ulcers and asymptomatic skin. All 10 comparisons have *P* values of <0.001. All box plots show medians with hinges corresponding to 25th and 75th percentiles and whiskers extending no further than 1.5× interquartile range from the hinges. Red diamonds signify means. Medians are shifted toward zero due to the absence of many of the ASVs in the majority of the samples. Abbreviations: AC, asymptomatic control specimens; CU, cutaneous ulcer specimens. Download FIG S1, PDF file, 1.3 MB. Copyright © 2021 Griesenauer et al. 2021 Griesenauer et al. This content is distributed under the terms of the Creative Commons Attribution 4.0 International license.

Next, we compared the 10 most abundant ASVs in the CU and AC microbiomes. The most abundant ASVs in the CU microbiome were Streptococcus pyogenes (11.01%), T. pallidum subsp. *pertenue* (8.34%), a *Fusobacterium* species (6.04%), Campylobacter sputorum (5.63%), Staphylococcus aureus (5.56%), three different H. ducreyi ASVs (4.91%, 4.39%, and 3.03%), Corynebacterium diphtheriae (3.56%), and Treponema denticola (1.56%) (Fig. 1C; Fig. S1C). Of these specific ASVs, only the S. aureus ASV was detected in the AC microbiome (0.92%). The most abundant ASVs in the AC microbiome were two Staphylococcus arlettae ASVs (3.95% and 1.29%), Micrococcus luteus (2.59%), Staphylococcus edaphicus (1.70%), Kytococcus sedentarius (1.58%), Brevundimonas nasdae (1.33%), Nocardioides lianchengensis (1.22%), Knoellia flava (1.10%), a *Deinococcus* species (1.07%), and a *Pseudonocardia* species (0.28%) (Fig. 1D; Fig. S1D). None of the top 10 AC microbiome ASVs were detected in the CU microbiome. Thus, the CU microbiome is enriched in bacterial species either not found or found in very low abundance in the AC microbiome and is less rich and diverse than the AC skin microbiome.

### Ulcer microbiome by etiology.

**FIG 2:**
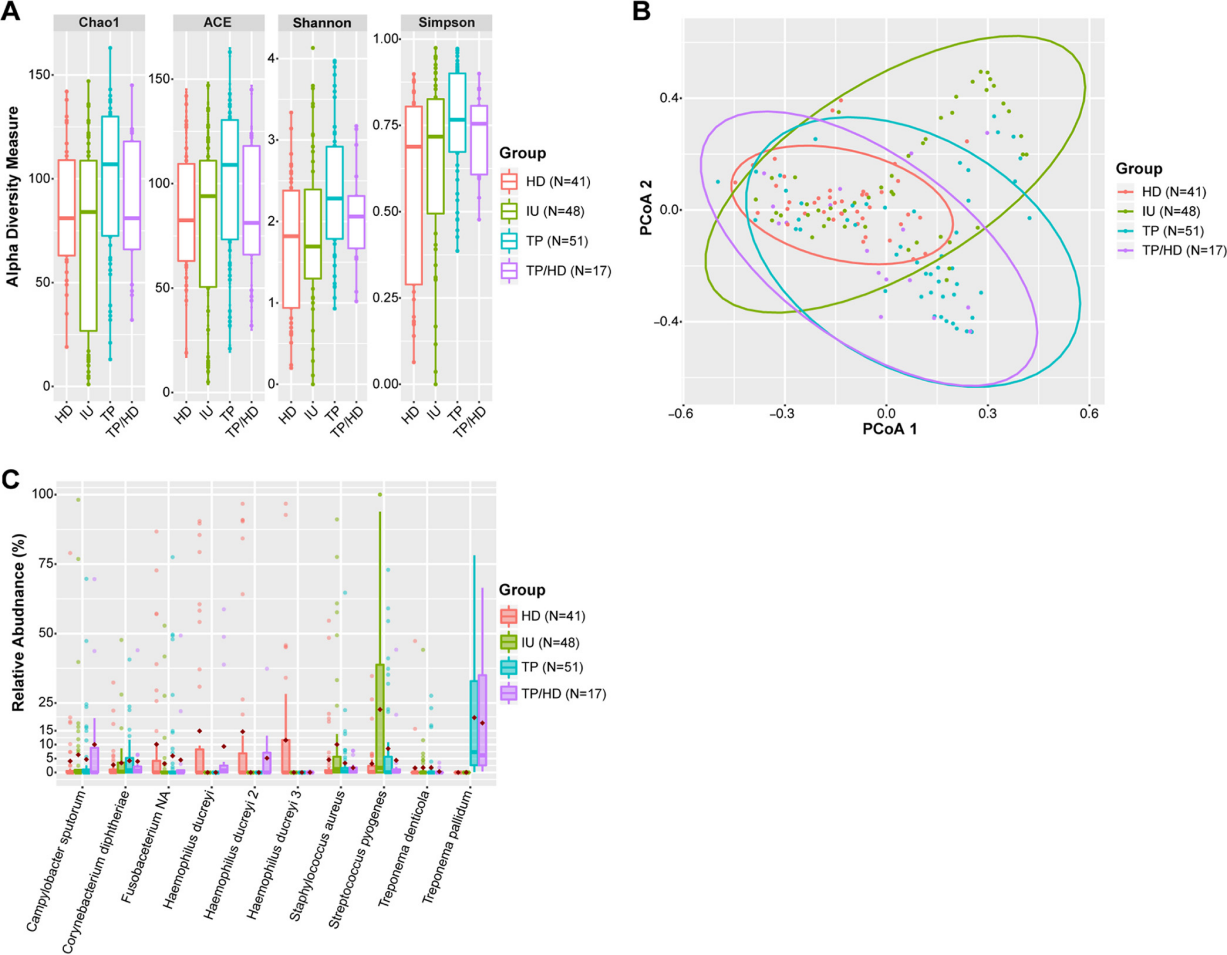
IU vs TP, HD, and TP/HD microbiomes in the stringent data set. (A) Box plot showing Chao1 and ACE richness estimates and Shannon and Simpson diversity indices of the different ulcer groups. Significant *P* values: Chao1, TP vs IU, 0.043; Shannon, TP vs HD, 0.002; TP vs IU, 0.018; Simpson, TP vs HD, 0.018. (B) PCoA of microbiomes by ulcer etiology. Significant pairwise PERMANOVA *P* values: TP vs HD, 0.002; TP vs IU, 0.002; HD vs IU, 0.002; HD vs TP/HD, 0.002, IU vs TP/HD, 0.002. (C) Box plots of top 10 ASVs found in ulcers and comparison of their relative abundances between all ulcer etiologies. Box plots show medians with hinges corresponding to 25th and 75th percentiles and whiskers extending no further than 1.5× interquartile range from the hinges. Red diamonds signify means. Medians are shifted toward zero due to the absence of many of the ASVs in the majority of the samples. Significantly different groups and *P* values follow: for Treponema pallidum, IU vs TP, *P* < 0.001; IU vs TP/HD, *P* < 0.001; TP vs HD, *P* < 0.001; HD vs TP/HD, *P* < 0.001; for Streptococcus pyogenes, IU vs HD, *P* < 0.001; IU vs TP, *P* = 0.037; IU vs TP/HD, *P* = 0.006; HD vs TP, *P* = 0.008; for Haemophilus ducreyi, IU vs HD, *P* < 0.001; IU vs TP/HD, *P* < 0.001; HD vs TP, *P* < 0.001; HD vs TP/HD, *P* = 0.043; TP vs TP/HD, *P* < 0.001; for Staphylococcus aureus, IU vs TP, *P* = 0.047; IU vs TP/HD, *P* = 0.047; for Haemophilus ducreyi 2, HD vs TP, *P* = 0.014; for Haemophilus ducreyi 3, HD vs TP, *P* < 0.001; HD vs TP/HD, *P* = 0.002. Abbreviations: HD, H. ducreyi ulcers; IU, idiopathic ulcers, TP, Treponema pallidum subspecies *pertenue* ulcers; TP/HD, dual positive ulcers.

As almost a third of the CU samples had no discernible etiology and were classified as IU (Table 2), we next determined which ASVs were present in IU and how they differed from the other CU groups. TP ulcers were richer (contained more bacterial species) and more diverse than HD and IU ulcers, but not the TP/HD ulcers, in the stringent data set (Fig. 2A). These effects were not evident in the overall data set (Fig. S2A). PCoA using Bray-Curtis dissimilarities showed that each CU microbiome was unique, with the IU microbiome possibly being the most distinct compared to the other groups (Fig. 2B; Fig. S2B).

10.1128/mBio.03162-20.5 FIG S2 IU vs TP, HD, and TP/HD microbiomes in the overall data set. (A) Box plots showing Chao1 and ACE richness estimates and Shannon and Simpson diversity indices. Significant *P* values: Shannon, TP vs HD, *P* = 0.049, TP vs IU, *P* = 0.049. (B) PCoA of microbiomes by ulcer etiology. Significant pairwise-PERMANOVA *P* values: TP vs HD, *P* = 0.002; TP vs IU, *P* = 0.002; TP vs TP/HD, *P* = 0.013; HD vs IU, *P* = 0.012; HD vs TP/HD, *P* = 0.013; IU vs TP/HD, *P* = 0.002. (C) Box plots of top 10 ASVs found in ulcers and comparison of their relative abundances between all ulcer etiologies. Box plots show medians with hinges corresponding to 25th and 75th percentiles and whiskers extending no further than 1.5× interquartile range from the hinges. Red diamonds signify means. Medians are shifted toward zero due to the absence of many of the ASVs in the majority of the samples. Significantly different groups and *P* values: for Treponema pallidum, IU vs TP, *P* < 0.001; IU vs TP/HD, *P* < 0.001; TP vs HD, *P* < 0.001; HD vs TP/HD, *P* < 0.001; for Streptococcus pyogenes, IU vs HD, *P* = 0.021; IU vs TP, *P* = 0.007; IU vs TP/HD, *P* < 0.001; HD vs TP/HD, *P* = 0.036; for Haemophilus ducreyi, IU vs HD, *P* < 0.001; IU vs TP, *P* = 0.004; IU vs TP/HD, *P* < 0.001; HD vs TP, *P* <0.001; TP vs TP/HD, *P* < 0.001; for Haemophilus ducreyi 3, IU vs TP, *P* < 0.001; IU vs TP/HD, *P* = 0.007; HD vs TP, *P* < 0.001; HD vs TP/HD, *P* = 0.007. Abbreviations: HD, H. ducreyi ulcers; IU, idiopathic ulcers, TP, Treponema pallidum subsp. *pertenue* ulcers; TP/HD, dual positive ulcers. Download FIG S2, PDF file, 1.0 MB. Copyright © 2021 Griesenauer et al. 2021 Griesenauer et al. This content is distributed under the terms of the Creative Commons Attribution 4.0 International license.

We next examined which ASVs were more abundant in IU than in TP, HD, and TP/HD. An ASV representing S. pyogenes was the most abundant in IU and was enriched in IU compared to the other CU subgroups (22.65% in IU versus [vs] 3.16%, 8.58%, and 4.38% for HD, TP, and TP/HD, respectively, in the stringent data set with similar numbers in the overall data set) (Fig. 2C; Fig. S2C). The relative abundance of S. aureus was also enriched in IU compared to the TP and TP/HD ulcers (10.06% vs 3.39% or 1.68%, respectively), but not the HD ulcers (10.06% vs 4.58%). These differences were observed only in the stringent data set. No other ASVs with a relative abundance over 1% were significantly different in IU compared to the other CU subgroups. These data suggest that the IU microbiome has a different bacterial composition, is enriched in S. pyogenes compared to other CU ulcers, and is enriched in S. aureus compared to TP and TP/HD ulcers but not HD ulcers.

### IU specimens without detectable S. pyogenes.

S. pyogenes was detected in 26 of 48 (∼54%) of IU specimens. Since T. pallidum subsp. *pertenue* was once thought to be the sole cause of CU and further investigation revealed the association of CU with H. ducreyi, we investigated the hypothesis that IU could also be a polymicrobial syndrome. We divided the IU specimens into subgroups based upon detection of S. pyogenes reads. Although the alpha diversity indices of the IU subgroups were similar (Fig. S3A), PCoA revealed that the two groups contained distinct bacterial communities (Fig. S3B). The relative abundances of C. sputorum (13.40% vs 0.46%) and three different ASVs of Catonella morbi (7.37% vs 0.16%, 5.36% vs 0.09%, and 4.29% vs 0.07%) were significantly higher in the S. pyogenes-negative subgroup compared to the S. pyogenes*-*positive subgroup (Fig. S3C). These results are consistent with the hypothesis that more than one bacterial species may be capable of causing IU.

10.1128/mBio.03162-20.6 FIG S3 IU classified by the absence of S. pyogenes. (A) Box plots of Chao1 and ACE richness estimates and Shannon and Simpson diversity indices. No significant differences were found. (B) PCoA of microbiomes by ulcer etiology. PERMANOVA *P* value = 0.001. (C) Box plots of top 10 ASVs found in IU and comparison of their relative abundances between IU with or without S. pyogenes. Box plots show medians with hinges corresponding to 25th and 75th percentiles and whiskers extending no further than 1.5× interquartile range from the hinges. Red diamonds signify means. Medians are shifted toward zero due to the absence of many of the ASVs in the majority of the samples. Significantly different ASVs and *P* values: Streptococcus pyogenes, *P* < 0.001; Campylobacter sputorum, *P* = 0.001; Catonella morbi, *P* = 0.006; Catonella morbi 2, *P* = 0.002; Catonella morbi 3, *P* = 0.001. Download FIG S3, PDF file, 0.7 MB. Copyright © 2021 Griesenauer et al. 2021 Griesenauer et al. This content is distributed under the terms of the Creative Commons Attribution 4.0 International license.

### “Improved” vs “not improved” follow-up ulcers.

**FIG 3:**
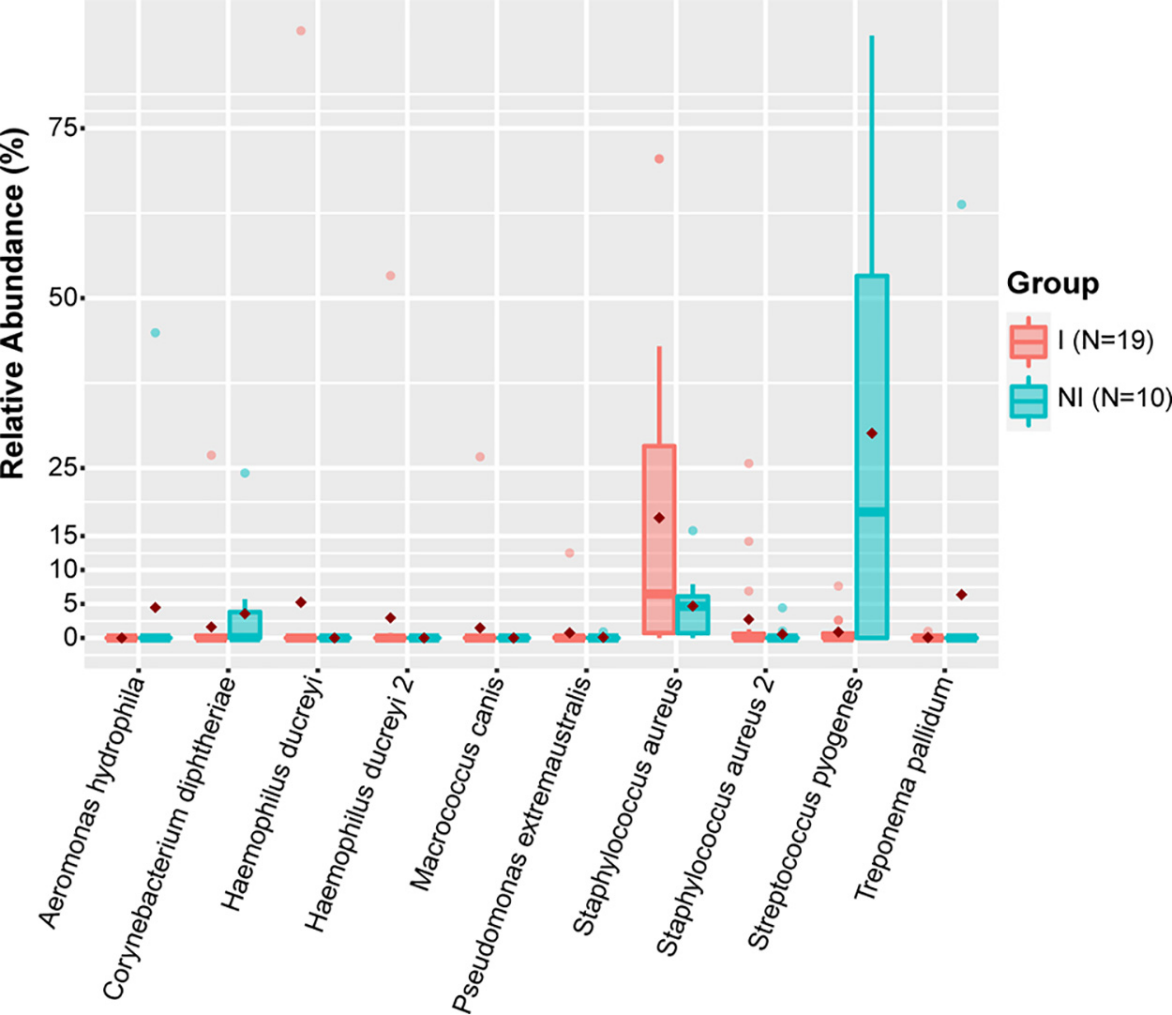
“Improved” and “not improved” ulcers. Box plots of top 10 ASVs found in follow-up ulcers and comparison of their relative abundances between “improved” (I) (*n* = 18) and “not improved” (NI) (*n* = 10) samples. Box plots show medians with hinges corresponding to 25th and 75th percentiles and whiskers extending no further than 1.5× interquartile range from the hinges. Red diamonds signify means. Medians are shifted toward zero due to the absence of many of the ASVs in the majority of the samples. Significantly different ASVs and *P* values: for Streptococcus pyogenes, *P* < 0.001, for Staphylococcus aureus, *P* = 0.005; for Treponema pallidum, *P* = 0.030.

Although rarely reported for HD, macrolide resistance occurs in both TP and S. pyogenes (13–15). To examine whether either TP or S. pyogenes might be associated with lack of improvement of CU in children treated with azithromycin, we determined the top 10 ASVs in the “improved” vs “not improved” follow-up ulcer groups. The “not improved” group had an average relative abundance of S. pyogenes of 30.11%. The relative abundance of S. pyogenes in the “improved” group was only 0.88%, or ∼34 times lower than the “not improved” group (Fig. 3); these differences were highly statistically significant (*P* < 0.001). TP was also enriched in the “not improved” group compared to the “improved” group with relative abundances of 6.38% and 0.06%, respectively. We did not find a difference in the average relative abundance of any of the HD ASVs in the top 10 ASVs between the two groups. S. aureus was found in higher relative abundance in the “improved” group compared to the “not improved” group (17.67% vs 4.69%, respectively). We detected *C. morbi* in only a single follow-up sample and did not detect *C. sputorum* in any of the follow-up samples. These observations are consistent with the hypothesis that macrolide-resistant TP and S. pyogenes could cause azithromycin treatment failures of CU, while HD, *C. morbi*, and *C. sputorum* are not associated with azithromycin treatment failures of CU.

### Absolute abundance of S. pyogenes.

IU had a higher relative abundance of S. pyogenes compared to that of other CU groups, so we hypothesized that the absolute abundance of S. pyogenes would also be higher in IU specimens. We performed quantitative PCR (qPCR) using S. pyogenes-specific *speB* (*Streptococcus* pyrogenic exotoxin B), which has been used to identify S. pyogenes from clinical samples (16), and human *β-actin* primers on all of the CU specimens. The copy numbers of *speB* and *β-actin* were calculated. As most of the DNA (95% to 99%) in the ulcer swabs is of host origin and the amount of DNA varied from specimen to specimen, we normalized *speB* copy number to *β-actin* copy number as a surrogate for unequal input DNA. We detected *speB* DNA in 100/157 samples, similar to the number of samples in which we detected S. pyogenes using 16S rRNA gene sequencing (93/157). The average absolute abundance of S. pyogenes in the IU microbiome was nine times higher than that in the HD or TP/HD microbiomes and twice as high than that in the TP microbiome. We did not test for the absolute abundance of *C. morbi* due to exhaustion of CU samples.

**FIG 4:**
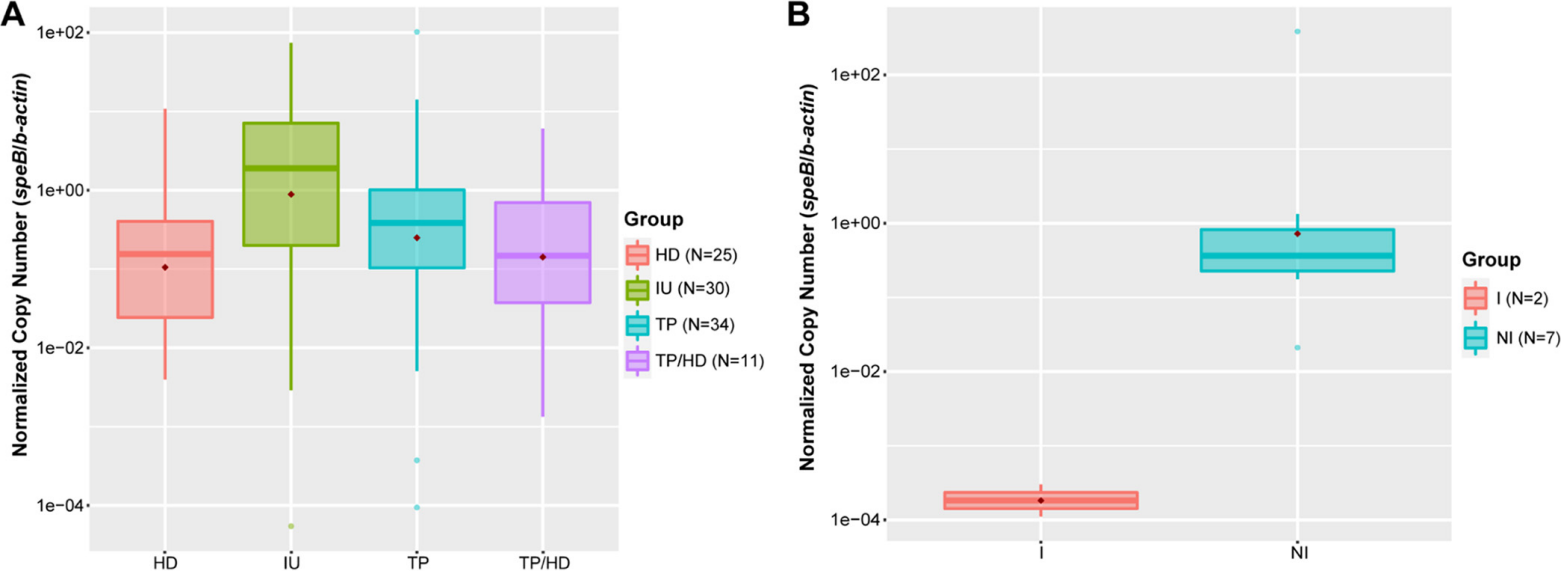
Absolute abundance of S. pyogenes in ulcers. (A) Box plot comparing estimated absolute abundances of S. pyogenes gene *speB* normalized to human *β-actin* in the stringently classified CU data set. N is the number of samples within each group in which a copy number could be calculated. IU vs HD, *P* = 0.056; all other *P* values do not approach significance. (B) Box plot comparing estimated absolute abundances of S. pyogenes gene *speB* normalized to human *β-actin* in “improved” vs “not improved” follow-up ulcers. N is the number of samples within each group in which a copy number could be calculated. *P* = 0.0004. All box plots show medians with hinges corresponding to 25th and 75th percentiles and whiskers extending no further than 1.5× interquartile range from the hinges. Red diamonds signify means. Abbreviations: HD, H. ducreyi ulcers; IU, idiopathic ulcers; TP, Treponema pallidum subsp. *pertenue* ulcers; TP/HD, dual positive ulcers.

Because S. pyogenes had a higher relative abundance in the “not improved” follow-up ulcers compared to that in the “improved” follow-up ulcers, we hypothesized that the absolute abundance would show similar results. The average absolute abundance of S. pyogenes in the “not improved” group was ∼270,000 times higher than that in the “improved” group by our S. pyogenes-specific qPCR assay (Fig. 4B). These data show that S. pyogenes is enriched in the IU microbiome compared to the TP, HD, and TP/HD microbiomes and that S. pyogenes is more abundant in the “not improved” microbiome compared to the “improved” microbiome. These data also further strengthen the hypothesis that azithromycin treatment failures of CU can be mediated by macrolide-resistant S. pyogenes.

## DISCUSSION

The WHO estimates that nearly one-third of CU in yaws-endemic areas are idiopathic (10, 17). To identify possible bacterial agents of IU, we performed V1-V3 16S rRNA gene sequencing on CU samples that had been identified as positive for TP, HD, both, or neither based on PCR testing, as well as on community-matched asymptomatic skin controls, environmental controls, and follow-up ulcer samples. We found that a single ASV of S. pyogenes was highly enriched in IU and in ulcers that had not improved after treatment with azithromycin, suggesting a causal role for this organism in CU.

Microbiome samples from an infected site and a similar contralateral site from the same person are more alike than a microbiome sample from an infected site on one person and from the same site on a noninfected person (18). Therefore, we sampled healthy school or household members as AC rather than intact skin of an infected individual by swabbing their lower leg, as skin microbiomes are largely influenced by the microenvironment of the sampled site (19). Premoistened swabs are considered superior to dry swabs in skin microbiome analysis (20); however, as this study was added onto an ongoing study that used dry swabs to sample CU, we used dry swabs for the AC samples. Thus, our sample collection method likely underestimated the diversity of bacteria from the samples, especially that from AC. For best coverage of skin bacterial communities, we sequenced the V1-V3 region, which provides results similar to that using shotgun sequencing for skin microbiomes (21).

We found that the AC microbiome samples were much more diverse than the CU microbiome samples. A diverse skin microbiome is hypothesized to promote health by preventing invading microorganisms from causing infection directly or by educating T cells in the skin to respond to specific microorganisms (19). In contrast, the diversity of the skin microbiome can be reduced during cutaneous infections, reflecting overgrowth of specific pathogens or opportunistic commensals, and possibly microorganisms that are more resistant to the host immune responses (12, 22, 23). Our observed alpha and beta diversity measurements for AC and CU samples are similar to previous studies that have compared microbiomes of healthy and diseased skin (12, 22, 23).

16S rRNA gene sequences cannot distinguish between T. pallidum subspecies. We therefore amplified and sequenced a T. pallidum subsp. *pertenue* positive control with the CU specimens The ASV of the positive control and the ASV corresponding to T. pallidum in the CU specimens were identical. Coupled with the fact that the PCR tests showed that the CU specimens contained T. pallidum subsp. *pertenue* DNA, the data suggest that the T. pallidum ASV in the CU specimens is likely of the subspecies *pertenue*.

Although each of the pathogen-associated CU groups contained a high mean relative abundance of the 16S rRNA gene reads of their respective pathogen(s), only 79% of the TP, HD, or dual positive samples, as classified by PCR, contained the expected 16S rRNA gene pathogen reads. Conversely, of the IU samples, over half contained low levels of TP and HD 16S rRNA gene reads. PCR has a higher sensitivity than 16S rRNA gene sequencing, but PCR can give a false-positive result due to environmental contamination, while 16S rRNA gene sequencing can assign abundant sequences to the wrong specimens due to errors in the bar codes. Our attempt at removal of environmental contaminations may have obscured low but real amounts of pathogen 16S rRNA gene. We did find low levels of HD in ∼21% of our AC samples before contamination removal, which agrees with the level of detection by PCR of HD on the skin of asymptomatic children reported in the area (9). In addition, we initially performed PCR tests on DNA isolated from the lysis buffer; if the results showed no HD or TP amplification, we concentrated the DNA samples and performed PCR a second time. We made the 16S rRNA gene libraries from unconcentrated samples only. Given the technical differences between the assays, we developed the parameters outlined in Table 2 to stringently classify CU samples. We performed all downstream analyses on both the stringent (*n* = 157) and overall (*n* = 265) data sets; the major results were similar in both data sets.

In the IU group in both data sets, an S. pyogenes ASV had the highest relative abundance and was three to seven times higher than that in other CU groups; the average relative and absolute abundances of S. pyogenes were higher in ulcers that had not improved following azithromycin treatment compared to those that had improved. S. pyogenes is a Gram-positive bacterium primarily found as part of the normal nasopharyngeal flora in 2 to 21% of healthy children (24, 25). In addition to causing pharyngitis, S. pyogenes can cause multiple skin infections, including impetigo, erysipelas, cellulitis, and rarely necrotizing fasciitis (26). In impetigo, S. pyogenes colonizes intact skin, especially on the legs, up to 10 days before disease onset and likely enters the skin via minor trauma (27). We did not detect S. pyogenes in the AC samples, suggesting that either transient colonization of the skin was uncommon, our methods were insufficient to detect colonization, or our contaminant removal protocol was too strict and excluded low but real S. pyogenes reads. Impetigo is the predominant form of S. pyogenes infection in the South Pacific (28), but S. pyogenes has not been described as a cause of CU. In many acute infections, a single pathogen outgrows the normal flora and causes disease; our data suggest that S. pyogenes is a causal agent of IU.

Humans are the only known reservoirs for S. pyogenes (29); cases of S. pyogenes skin infection total over 111 million per year (30). Cuts and abrasions on the skin are common, and S. pyogenes is found worldwide, yet S. pyogenes has not been associated with development of CU until this study. We have two hypotheses as to why we observed an outgrowth of S. pyogenes. (i) This is a new clade of S. pyogenes that is capable of causing skin ulcers. (ii) TP and/or HD could have initially caused formation of an ulcer, were cleared or had their abundances reduced due to pressure from azithromycin treatment and/or an active immune response, and S. pyogenes opportunistically colonized the ulcer and prevented ulcer healing. Isolation and whole-genome sequencing of S. pyogenes from IU are necessary to distinguish which hypothesis is more likely.

S. pyogenes can also become macrolide resistant (14, 15) and develop or acquire macrolide resistance through multiple mechanisms, including efflux through *mefA* (31), ribosomal methylation of the 50S ribosomal subunit by *ermB* or *ermTR* (32) or seldomly via mutations in the 23S rRNA gene and in the ribosomal protein L4 (33). As macrolide use is associated with the emergence of macrolide-resistant S. pyogenes (34, 35), we hypothesize that macrolide-resistant S. pyogenes may be present in the “not improved” ulcers. Culture-dependent studies and antimicrobial resistance testing are needed to address this hypothesis. Alternatively, shotgun sequencing could identify macrolide resistance genes in S. pyogenes from IU and “not improved” ulcers.

Approximately 45% of the IU samples did not contain detectable S. pyogenes 16S rRNA gene sequences, suggesting that there are additional causes of IU. We identified a *C. sputorum* ASV and three *C. morbi* ASVs that were enriched in IU that did not contain S. pyogenes. *C. sputorum* is a Gram-negative bacterium containing three biovars: faecalis, paraureolyticus, and sputorum. Only biovars paraureolyticus and sputorum are known to cause disease in humans (36). Both biovars have been found in human diarrheal samples, while only biovar sputorum has been found in the oral cavity and in abscesses (37, 38). Our 16S rRNA gene sequencing could not differentiate between biovars. *C. morbi* is a Gram-negative bacterium that has been isolated only in the gingival crevices of humans and is associated with oral disease (39–41). Similar to S. pyogenes, *C. sputorum* and *C. morbi* were not detected in the AC samples. The relative abundance for *C. sputorum* was similar between IU and the other CU groups, while *C. morbi* was found predominately in IU samples; these data suggest that *C. sputorum* can grow in CU in the presence of TP and HD but *C. morbi* cannot. Both *C. sputorum* and *C. morbi* were found in very low relative abundance in S. pyogenes-associated IU, suggesting that S. pyogenes can outcompete both species. We hypothesize that *C. morbi* may be a secondary agent of IU in which S. pyogenes is absent and that *C. sputorum* more likely thrives in the environment of CU. Whether *C. morbi* is directly causing IU or colonizing an already present ulcer is unclear. *C. sputorum* was not found in follow-up ulcers and one *C. morbi* ASV, which was not one of the three ASVs enriched in IU, was detected in a single follow-up ulcer at a low abundance, suggesting that these bacteria are not azithromycin resistant, consistent with limited previous reports (41, 42).

S. pyogenes, *C. morbi*, and *C. sputorum* can all be members of the human oral microbiome, and *C. sputorum* has been isolated from the feces of swine with proliferative enteritis (42). Given that we are detecting these bacteria in CU, these oral or fecal organisms may be reaching the skin and causing CU or colonizing already formed CU. The study was done on Lihir Island, a rural area; according to the Papua New Guinea government’s Water, Sanitation, and Hygiene Policy 2015 to 2030, only 13% of the rural population has access to basic sanitation (43). In Papua New Guinea, the chewing and spitting of betel nuts are a major health concern and are banned in the capital city, Port Moresby. On Lihir Island, livestock, including pigs, are not fenced and walk freely among the villagers, betel nut chewing is common, and children walk barefoot and play on the ground. Spitting on the ground where children play, exposure to animal feces, and the lack of access to basic sanitation and hygiene could allow oral or fecal flora to reach the lower legs of these children and cause or colonize ulcers.

An S. aureus ASV was the second most relative abundant ASV detected in IU, but was only enriched compared to the TP and TP plus HD groups in the stringent data set, and was enriched in CU compared to AC. S. aureus is a Gram-positive bacterium that is a component of the normal skin flora (44). Although usually a commensal, S. aureus can invade the skin and cause impetigo, folliculitis, furuncles, and subcutaneous abscesses (45). S. aureus is known to colonize wounds and can inhibit or delay wound healing (46–48). S. aureus was enriched in the “improved” ulcer group compared to the “not improved” ulcer group. Given that S. aureus was not consistently enriched in IU compared to other groups and that S. aureus was enriched in “improved” ulcers compared to “not improved” ulcers, S. aureus is likely colonizing the ulcers and not driving ulcer formation.

A *Fusobacterium* species, later identified as F. ulcerans, has been implicated in CU (49). A *Fusobacterium*, unidentified at the species level, was our third most abundant ASV in CU; however, there was no difference in relative abundance between CU subgroups. The same strains of *F. ulcerans* were found in the mud from areas where people had CU (50). If the *Fusobacterium* species we identified proves to be the same *F. ulcerans* found in the soil, it may simply be colonizing an already formed ulcer, regardless of ulcer cause.

While our overall data set and stringent data set showed similar major findings, we did observe some minor differences. Our TP ulcers showed higher alpha diversity compared to IU and HD ulcers in the stringent data set but not the overall data set. If TP ulcers really do have a higher alpha diversity, we hypothesize that TP is changing the microenvironment less than HD or the causative agent of IU, thus allowing more flora to survive in the ulcer microenvironment. We also observed that the relative abundance of S. aureus was significantly enriched in IU compared to TP and TP/HD in the stringent data set but not in the overall data set. If S. aureus is enriched in IU compared to TP and TP plus HD, we hypothesize that TP is either directly inhibiting S. aureus growth or is likely creating a microenvironment that is not conducive to S. aureus growth.

Shotgun sequencing was performed on samples obtained earlier (October 2013 to October 2014) during the yaws eradication campaign on Lihir Island to identify agents of IU (51). S. pyogenes and S. aureus were detected in CU in that study (51); however, their relative abundances were much lower than what we found in CU in this study (<1% for both S. pyogenes and S. aureus in the shotgun sequencing study vs 11.01% and 5.56%, respectively, in this study). The shotgun sequencing study identified Corynebacterium diphtheriae, Arcanobacterium haemolyticum, and Streptococcus dysgalactiae as possible agents of IU; while we also found these bacterial species in IU (3.45%, <0.5%, and 1.13%, respectively), we did not find any difference between any of these species in IU versus that in other CU groups. The discrepancies between the studies may be due to the different sample collection periods or technical differences between the studies. (i) The sequencing depth was different between the studies (678 median reads with species level identification in the CU samples in the shotgun sequencing study versus 42,481 median reads with species level identification in the CU samples in this study). (ii) This study’s ability to account for environmental contamination, which the previous study could not as their controls contained insufficient DNA for shotgun sequencing. (iii) 16S rRNA gene primers identify bacteria only, while shotgun sequencing identifies other microbes as well, which could inflate the relative abundance that we observed compared to the previous study.

A limitation of this study and most microbiome studies is that we cannot prove causation between a bacterium identified through 16S rRNA gene sequencing and IU; we can claim only an association. 16S rRNA gene sequencing involves preamplification before sequencing, and even the best 16S rRNA gene primers have bias toward certain bacterial species (52, 53). Thus, it is possible that our data may overestimate or underestimate relative abundances of some species of bacteria. In this data set, we could not identify 30% of ASVs at the species level. Although the V1-V3 region of the 16S rRNA gene is the best region for skin bacterial identification (21), some V1-V3 regions may be too similar enough to discern different species of bacteria. A limited reference library may also explain why we were unable to identify some ASVs. We attempted to minimize this limit by using the Bayesian-based lowest common ancestor (BLCA) method to identify ASVs, which provides higher classification accuracy than existing tools (54).

This study presents the first survey of asymptomatic and diseased skin microbiota in a CU-endemic area. We identified S. pyogenes and *C. morbi* to be associated with IU and S. pyogenes to be associated with nonhealing CU on Lihir Island. Whether these bacteria are actual causes of ulcers or are a result of multimicrobial succession throughout the development and recovery from a skin ulcer is yet to be determined. Given that there have been no records of penicillin resistance for T. pallidum, S. pyogenes, or *C. morbi*, an attractive second line of therapy for nonhealing ulcers is benzathine penicillin. Future studies, including shotgun sequencing, are under way to confirm the association between S. pyogenes and IU, to identify other possible azithromycin-resistant bacteria present in CU that may delay ulcer healing, and to identify nonbacterial etiologies of CU.

## MATERIALS AND METHODS

### Participants.

The participants were part of a prospective cohort study in a yaws eradication campaign on Lihir Island, Papua New Guinea (7, 8). Participants with papillomatous skin lesions or atraumatic skin ulcers that measured >1 cm were eligible for the study. We analyzed cutaneous ulcer (CU) specimens obtained at 36, 42, and 48 months (May 2016, November 2016, and May 2017, respectively) after MDA. Demographic information of the participants with CU is summarized in Table S1 in the supplemental material.

Children who did not have ulcers but were present in the same classroom as the CU patient or were a family member served as AC (*n* = 233). Forty-two specimens in our study were collected from patients who presented with CU at the Lihir Medical Centre and therefore did not have a matched AC sample; AC samples were not collected for four CU participants sampled in the field. Informed written consent was obtained from all participants, or their parents or guardians, and verbal agreement from the children was obtained before enrollment. The protocol was approved by the National Medical Research Advisory Committee of the Papua New Guinea Ministry of Health (MRAC number 12.36) (7, 8).

### Clinical and environmental sample collection.

All CU specimens (*n* = 279) were collected as previously described (7). In brief, a sterile, dry, Dacron-tipped swab was vigorously rubbed at the base of the ulcer and inserted into an Eppendorf tube containing 0.5 ml lysis buffer (10 mM Tris-HCl, 0.1 M EDTA, 0.5% sodium dodecyl sulfate [SDS]). After treatment, participants with CU were examined 2 weeks later for ulcer healing. If an ulcer was still present, the participant had their ulcer swabbed again (*n* = 31). Follow-up ulcers were classified as improved if the ulcer bed was reduced by ≥50% of its diameter or ≥50% of the wound bed was filled by granulation tissue, and as not improved if there is < 50% diameter reduction, a nongranulating wound bed, or necrotic tissue. AC samples were obtained by swabbing a 5 × 5 cm area of intact lower leg skin and placing the dry swab into a tube containing lysis buffer. To account for possible environmental contamination, EC were collected in the presence of the CU patients (*n* = 234), the follow-up CU patients (*n* = 31), and the AC (*n* = 233) by waving a dry swab in the air and placing it in lysis buffer without touching the participant. To minimize laboratory contamination, we used dedicated lots of reagents, including Eppendorf tubes, swabs, and lysis buffer for each time point. All specimens were shipped to Indiana University Purdue University of Indianapolis and stored at −80°C. For the CU specimens, an aliquot of the lysis buffer was removed, and the remaining sample, including the swab, was shipped to the University of Washington (Seattle) for detection of HD or TP DNA by real-time PCR.

### PCR and qPCR.

To identify HD or TP DNA in ulcer samples, a real-time PCR was initially performed on the extracted DNA using a multiplex TaqMan assay, utilizing TaqMan MGB probes (Thermo Fisher Scientific, Carlsbad, CA, USA), with four or five replicates. We adapted the primer and probe sequences used in simultaneously detecting HD (targeting the 16S rRNA gene) and TpN47 (*tp0547*) of TP as described by Orle et al. (55), which detects all subspecies of TP. Specifically, we removed the biotin label in the primers and added a 6-carboxyfluorescein (FAM)-labeled reporter dye and a nonfluorescent quencher at the 3′ end for HD detection. We also designed a more sensitive TpN47 primer/probe set for simultaneous detection of both TP and HD organisms using ViiA7 Real Time PCR system (Thermo Fisher Scientific, Carlsbad, CA, USA). To detect TP, a forward primer sequence (5′-CAA GTA CGA GGG GAA CAT CG- 3′) and the reverse primer sequence 5′-TGA TCG CTG ACA AGC TTA GG-3′ were used to amplify a portion of TpN47. The TaqMan-MGB probe sequence for TP was 6′NED-CGG AGA CTC TGA TGG ATG CTG CAG TT-NFQMGB, where NED was the reporting dye and NFQ is a nonfluorescent quencher. The final concentration for all primers and probes was 0.5 μM in 1× TaqMan Fast Advanced Master Mix (Thermo Fisher Scientific, Carlsbad, CA, USA) per 10-μl reaction. The amplification profile is as follows: (i) 50°C for 2 min, (ii) 95°C for 30 s, and (iii) 45 cycles with each cycle consisting of 5 s at 95°C and 20 s at 60°C. A sample was considered positive for TP and/or HD if threshold cycle (*C_T_*) values were below 40 for at least one of the replicates amplified. The Tp548 gene was amplified by standard PCR as previously described (56). To distinguish between TP subsp. *pertenue* and TP subsp. *pallidum*, we amplified a unique molecular signature in the region of *tprL* (*tp1031*) (57) to differentiate the two subspecies using standard PCR, in which a 200-bp amplicon is observed for subspecies *pertenue* and a 600-bp product is seen for subspecies *pallidum*. The specific primers for amplifying this region of *tprL* are sense 5′-CTC TGC GCA CTG AGA ATT GCA-3′ and antisense 5′-GCA GTT CGG GTC CTT GCC AA-3′. Briefly, in a 50-μl reaction mixture, 5 μl of DNA was amplified using 2.5 U of GoTaq Flexi DNA polymerase, 200 nmol/liter deoxynucleoside triphosphate (dNTP), 0.6 mmol/liter primers, 1.5 mmol/liter MgCl_2_, and 1× GoTaq Flexi buffer (Promega, Madison WI). Cycling conditions were as follows: (i) 95°C for 5 min; (ii) 45 cycles, with each cycle consisting of 95°C for 1 min, 60°C for 1 min, and 72°C for 1 min; and (iii) 72°C for 10 min.

DNAs from samples that were initially negative for HD and/or TP were precipitated and concentrated, and PCR was repeated on the concentrated samples. As a DNA integrity control for specimens with no detectable HD or TP, we amplified CU specimens for human β-globulin DNA using the following primer sequence sets (forward [F], 5′-GAA GAG CCA AGG ACA GGT AC-3′ and reverse [R], 5′-CAA CTT CAT CCA CGT TCA CC-3′) with cycling conditions as follows: (i) 94°C for 3 min; (ii) 45 cycles with each cycle consisting of 94°C for 1 min, 60°C for 2 min, and 72°C for 1 min, and (iii) 72°C for 10 min. DNA was amplified using 2.5 U of GoTaq Flexi DNA polymerase, 200 nmol/liter dNTP, 0.5 mmol/liter primers, 1.5 mmol/liter MgCl_2_, and 1× GoTaq Flexi buffer (Promega, Madison WI) with an expected size of 268 bp.

Numerous no-template and DNA extraction negative controls were included with each PCR run, and were never positive, over several hundred samples. Thus, for the overall CU data set, if a single well from the unconcentrated or concentrated specimen yielded a positive result for HD or TP, the specimen was considered pathogen positive. For a specimen to be declared pathogen positive in the stringent data set, at least 75% of the wells from the unconcentrated specimens had to contain pathogen sequences.

To identify absolute abundance of S. pyogenes, we used primers specific for the S. pyogenes gene *speB* (16) and primers for human β-actin (F, 5′-GCT AAG TCC TGC CCT CAT TT-3′; R, 5′-GTA CAG GTC TTT GCG GAT GT-3′). Standard curves were generated using genomic DNA (gDNA) from a pure culture of S. pyogenes (ATCC 12384) and gDNA isolated from HeLa cells. qPCR for these experiments was performed in triplicate using the QuantiTect SYBR qPCR kit (Qiagen), according to manufacturer’s instructions, on an ep realplex4 Mastercycler (Eppendorf). qPCR cycling conditions consisted of an initial 15-min denaturing step at 95°C, followed by 40 cycles with each cycle consisting of 94°C denaturing for 15 s, 58°C annealing for 30 sec, and 72°C extending for 30 s. To determine whether only a single product was amplified, a melt curve analysis was added, transitioning from 60°C to 90°C at a rate of 0.5°C per second.

### 16S rRNA gene library preparation and sequencing.

We extracted DNA from the unconcentrated lysis buffer of the clinical and environmental control samples using the DNeasy blood and tissue kit (Qiagen) with pretreatment for Gram-positive bacteria, according to the manufacturer’s protocol, except we used 40 mg/ml lysozyme (Research Products International). DNA concentrations were quantified on a Qubit 4 fluorometer (Thermo Fisher Scientific) using the Quant-iT double-stranded DNA (dsDNA) assay kit, high sensitivity (Invitrogen). Samples were stored at 4°C until library construction.

Additional assay controls consisted of DNA isolation buffers, lysis buffers, and lysozyme or water used during 16S rRNA gene preparation (total *n* = 37). Positive controls consisted of an equal mixture of gDNA by weight of TP and HD or a mock community skin control (BEI Resources catalog no. HM-782D) (total *n* = 32).

Assay and positive controls were processed in parallel with the clinical samples. 16S rRNA gene libraries were made with the NEXTflex 16S V1-V3 Amplicon-Seq kit (Bioo Scientific Corp.), according to the manufacturer’s protocol, with an input DNA amount of 5 ng. To minimize batch effect, dedicated lots of reagents were used in 16S rRNA gene library preparation. Thirteen pools of ∼96 specimens were generated, with each pool having a mock community control, a TP/HD gDNA control, and assay controls to monitor data between pools. Pools were sequenced on an Illumina MiSeqDX platform using the 2 × 300 cycle kit at the Indiana University Medical Genomics Core, Indianapolis, Indiana. In all pools, the positive controls yielded the expected amplicons (data not shown). 

### Sequence processing and analysis.

Paired-end raw reads of 1,037 samples from 13 pools, including positive and negative controls, were processed using DADA2 pipeline v.1.8 with minor modifications (11). Specifically, reads were first trimmed at 290 bp and 285 bp for forward and reverse reads, respectively. Then, reads with higher than 10 expected errors were discarded. Each pool’s sequencing error rate was determined individually before merging. Chimeras were detected and removed using the default consensus method in the DADA2 pipeline. Taxonomy of the resultant 180,671 ASVs were identified with BLCA using the 16S rRNA database from NCBI (time stamped on 24 September 2019) with the addition of two extra species (54). Due to the incompleteness of the 16S rRNA database from NCBI, 14 HD and 4 TP distinct 16S rRNA sequences were manually added to the database. Taxonomy with a confidence score greater than 80 was considered trustworthy identification and was used for downstream analysis.

### Sequencing analyses and statistics.

Data were first pruned by removing samples with fewer than 500 total reads and removing taxa with fewer than 200 total reads in all samples. Amplicons detected in the EC and assay controls were removed from the clinical samples during the data enrichment step described in Results.

Analyses of ASV count tables were performed in the R statistical computing environment (58). We used the packages phyloseq (59) and vegan (60) to determine relative abundances, Bray-Curtis dissimilarities, ACE and Chao1 richness estimates, and Shannon and Simpson diversity indices. data.table (61), dplyr (62), and DataCombine (63) were used for data manipulation, and ggplot2 (64) was used for graphics. We used principal-coordinate analyses with Bray-Curtis dissimilarities to visualize differences among groups. Permutational multivariate analysis of variance (PERMANOVA) was used to determine differences in centroids among groups tested. To determine differences among alpha diversities, we used the Wilcoxon rank sum test for nonparametric comparisons. To determine differences among relative abundances of taxa between groups and to determine differences in S. pyogenes abundances among groups, we used gamma hurdle modeling using the log linker. We used Fisher’s exact test to test for possible confounding factors. The Benjamini and Hochberg procedure corrected for multiple comparisons with corrected *P* values of less than 0.05 considered significant.

### Data availability.

Raw sequences were deposited in the Sequence Read Archive with the accession numbers PRJNA663629 and PRJNA663631.
