# Supplementary material for: Streptococcus pyogenes Is Associated with Idiopathic Cutaneous Ulcers in Children on a Yaws-Endemic Island
Source: mBio. 2021 Jan 12;12(1):e03162-20. doi: 10.1128/mBio.03162-20 (PMC7844543; doi:10.1128/mBio.03162-20)
Supplement: TABLE S1 [file mBio.03162-20-st001.docx]

| **Table S1. Specimens and Demographic Information of Children with Ulcers** | | | | |
| --- | --- | --- | --- | --- |
| **Number of Specimens** | | | | |
| **Time post MDA** | **36 mo.** | **42 mo.** | **48 mo.** | **Total (%)** |
| Ulcers | 106 | 107 | 66 | 279 |
| Ulcer Environmental Controls^a^ | 103 | 82 | 49 | 234 |
| Asymptomatic Community Controls | 103 | 82 | 48 | 233 |
| Asymptomatic Environmental Controls | 103 | 82 | 48 | 233 |
| **Number of Follow-up Specimens^b^** | | | | |
| Improved | 10 | 7 | 3 | 20 |
| Not improved | 6 | 4 | 1 | 11 |
| Total | 16 | 11 | 4 | 31 |
| **Demographic Information^c^ of Children with Ulcers** | | | | |
| Male | 62 | 64 | 38 | 164 (59) |
| Female | 44 | 43 | 26 | 113 (41) |
| 1-5 yrs. | 15 | 18 | 7 | 40 (14) |
| 6-11 yrs. | 56 | 69 | 37 | 162 (59) |
| 12-17 yrs. | 26 | 14 | 15 | 55 (20) |
| 18+ yrs. | 9 | 6 | 5 | 20 (7) |
| ^a^, environmental controls were not collected from 45 participants  ^b^, 31 environmental controls were collected with the follow-up specimens  ^c^, demographic information missing on 2 participants | | | | |
