# Supplementary material for: Streptococcus pyogenes Is Associated with Idiopathic Cutaneous Ulcers in Children on a Yaws-Endemic Island
Source: mBio. 2021 Jan 12;12(1):e03162-20. doi: 10.1128/mBio.03162-20 (PMC7844543; doi:10.1128/mBio.03162-20)
Supplement: TABLE S2 [file mBio.03162-20-st002.docx]

| **Table S2. Summary of Sequencing Results after Pruning** | | | | | | | | |
| --- | --- | --- | --- | --- | --- | --- | --- | --- |
|  | **All (N=892)** | **CU (N=265)** | **AC (N=194)** | **EC (N=332)** | **Follow-Up (N=28)** | **Follow-Up EC (N=20)** | **Assay Control (N=21)** | **Positive Control (N=32)** |
| **Total Raw Reads** | 168,379,482 | 61,891,106 | 46,732,295 | 36,431,356 | 3,958,791 | 1,407,492 | 2,694,345 | 15,264,097 |
| **Mean Reads/Sample** | 188,555 | 233,551 | 240,888 | 109,733 | 136,510 | 70,375 | 128,302 | 477,003 |
| **Total High-Quality Reads** | 81,957,985 | 29,434,930 | 21,525,464 | 19,096,920 | 1,985,871 | 773,828 | 1,428,767 | 7,712,205 |
| **Mean High-Quality Reads/Sample** | 91,778 | 111,075 | 110,956 | 57,521 | 68,478 | 38,691 | 68,037 | 241,006 |
| **Percent High-Quality Reads/Sample** | 46.26% | 45.66% | 45.14% | 46.80% | 46.01% | 50.85% | 48.05% | 48.57% |
| Abbreviations: CU: Cutaneous ulcer; AC: Asymptomatic control; EC: Environmental control | | | | | | | | |
