# Supplementary material for: Streptococcus pyogenes Is Associated with Idiopathic Cutaneous Ulcers in Children on a Yaws-Endemic Island
Source: mBio. 2021 Jan 12;12(1):e03162-20. doi: 10.1128/mBio.03162-20 (PMC7844543; doi:10.1128/mBio.03162-20)
Supplement: TABLE S3 [file mBio.03162-20-st003.docx]

| **Table S3. 16S rRNA Sequencing Results Grouped by PCR Classification** | | | | | | | | |
| --- | --- | --- | --- | --- | --- | --- | --- | --- |
| **PCR Classification** | **HD by PCR** | | **TP by PCR** | | **TP/HD by PCR** | | **IU by PCR** | |
| **HD or TP Reads** | **HD** | **TP** | **HD** | **TP** | **HD** | **TP** | **HD** | **TP** |
| **Mean Read Count** | 24,548 | 5 | 464 | 7411 | 18,218 | 8717 | 602 | 7 |
| **Mean Relative Abundance** | 15.7% | < 0.1% | 0.2% | 10.8% | 13.7% | 7.0% | 1.0% | < 0.1% |
| **Number of samples positive by 16S rRNA \| Number of samples positive by PCR** | 63\|85 | 22\|85 | 52\|83 | 74\|83 | 30\|36 | 26\|36 | 37\|61^a^ | 17\|61^a^ |
| ^a^, Number for samples negative by 16S rRNA \| Number of samples negative by qPCR  Abbreviations: PCR – polymerase chain reaction; HD – *H. ducreyi*; TP – *T. pallidum* sub. *pertenue*;  TP/HD – *T. pallidum* sub. *pertenue* and *H. ducreyi*; IU – Idiopathic ulcer | | | | | | | | |
