# Supplementary figures and images for: Streptococcus pyogenes Is Associated with Idiopathic Cutaneous Ulcers in Children on a Yaws-Endemic Island
Source: mBio. 2021 Jan 12;12(1):e03162-20. doi: 10.1128/mBio.03162-20 (PMC7844543; doi:10.1128/mBio.03162-20)

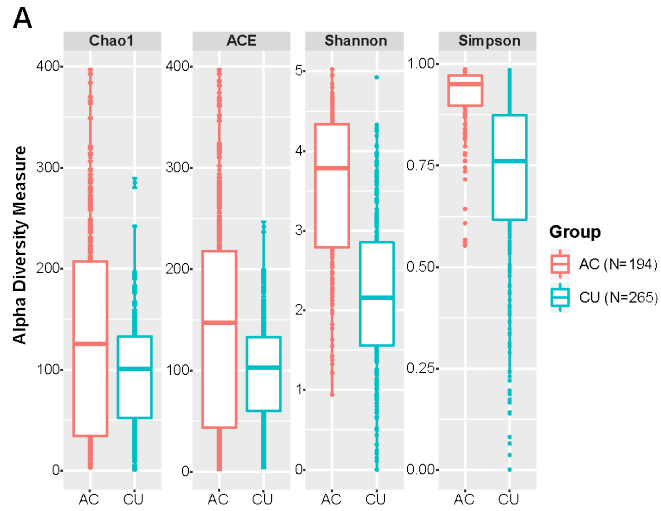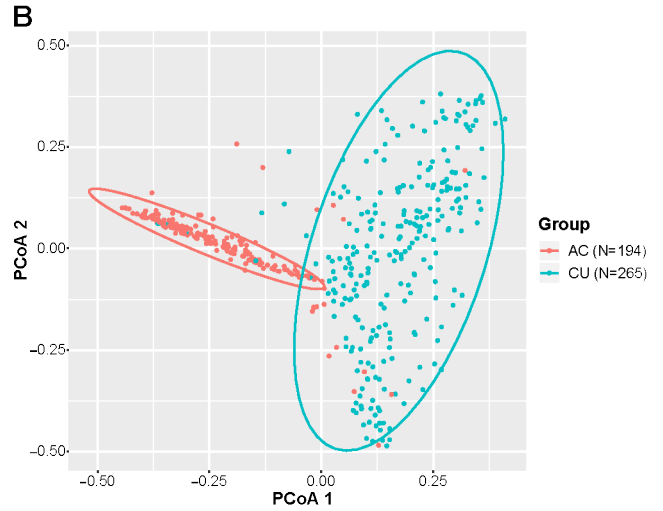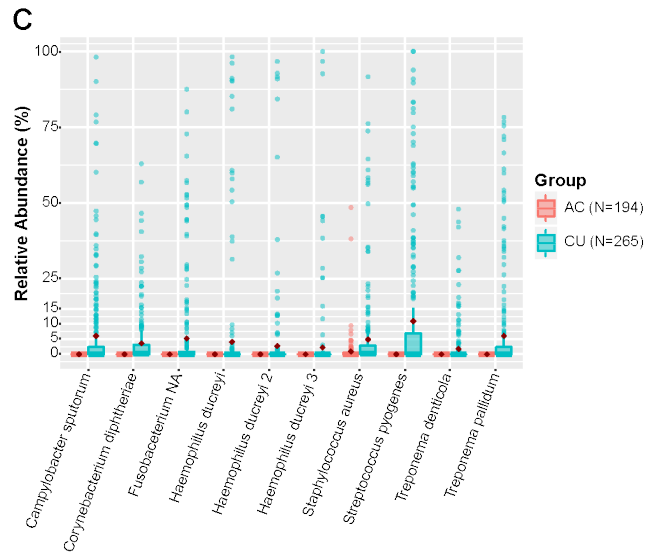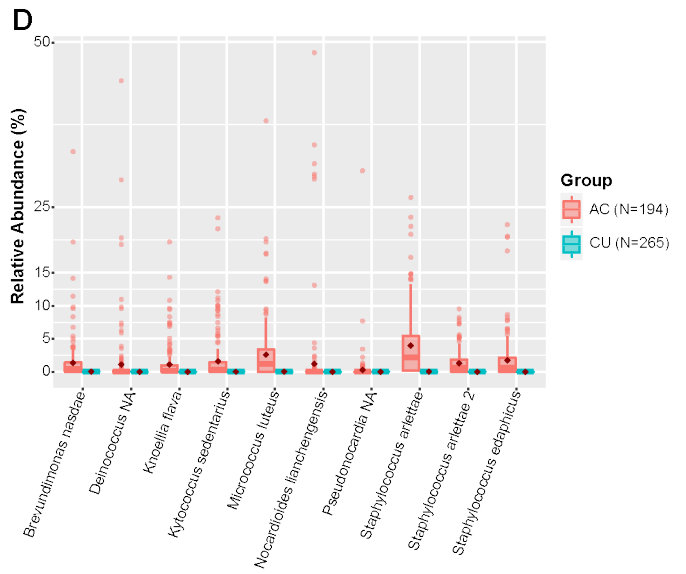

Supplement: FIG S1 [file mBio.03162-20-sf001.pdf]

A

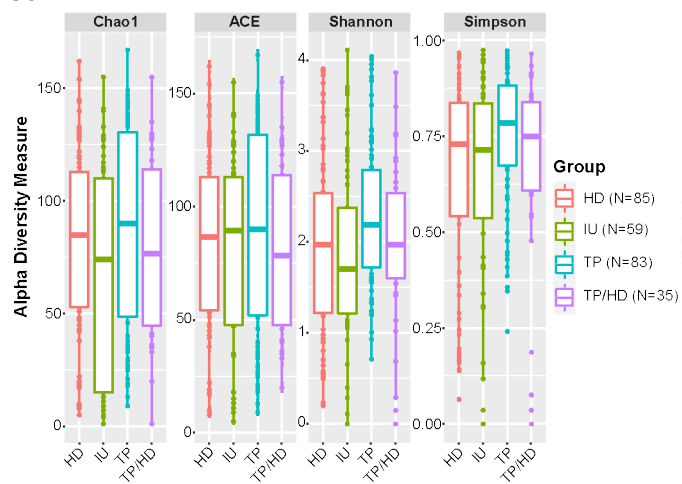

B

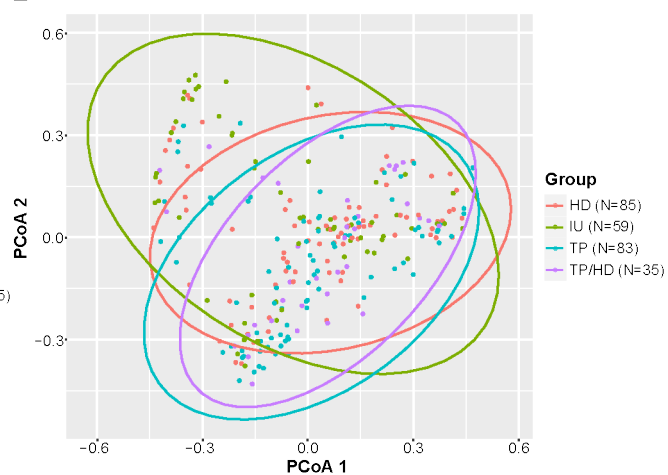

C

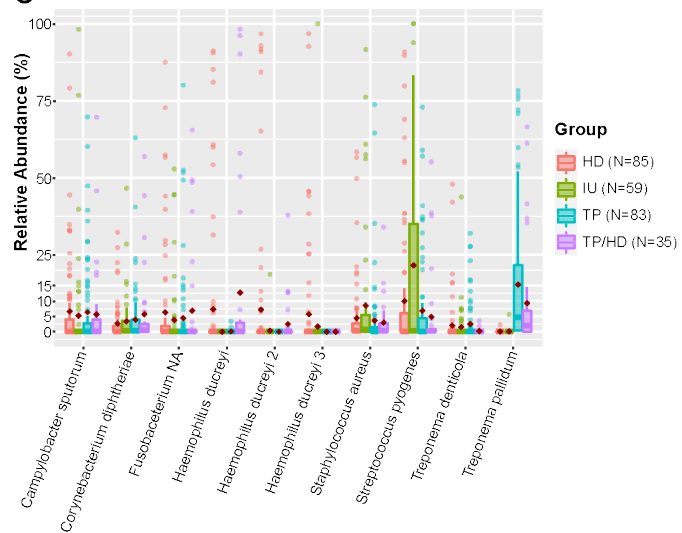

Supplement: FIG S2 [file mBio.03162-20-sf002.pdf]

**A**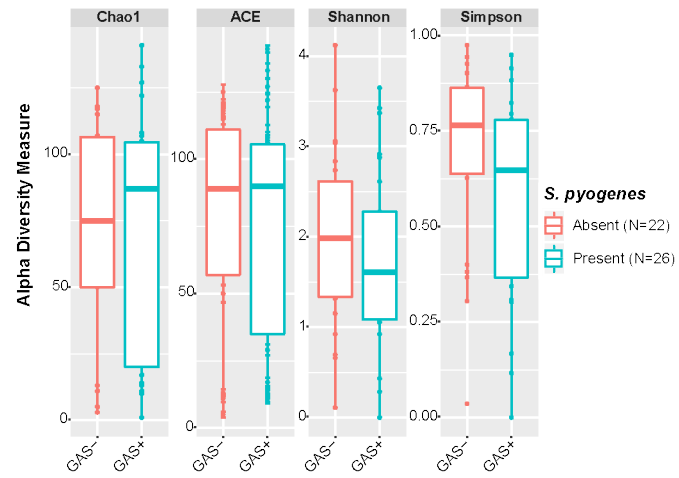**B**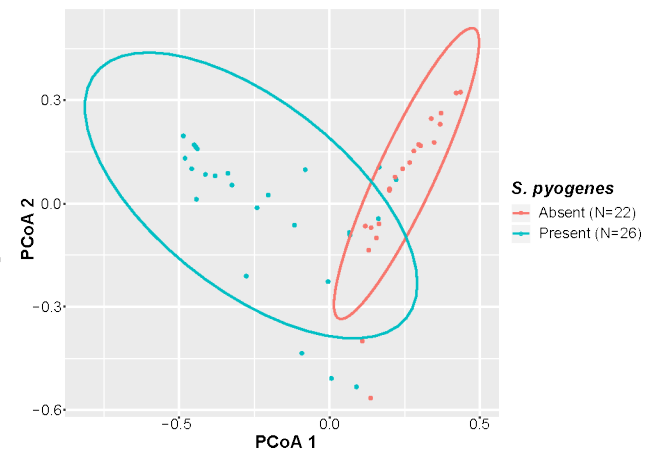**C**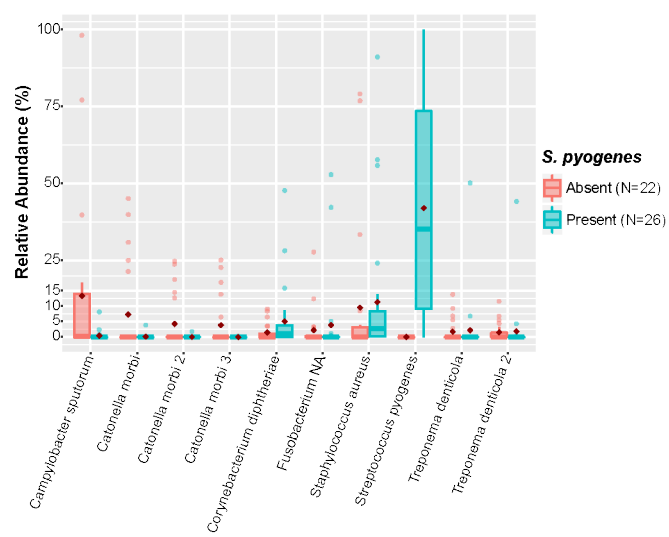

Supplement: FIG S3 [file mBio.03162-20-sf003.pdf]
